# Severe pediatric acute encephalopathy syndromes related to SARS-CoV-2

**DOI:** 10.3389/fnins.2023.1085082

**Published:** 2023-02-27

**Authors:** Hiroshi Sakuma, Jun-ichi Takanashi, Kazuhiro Muramatsu, Hidehito Kondo, Takashi Shiihara, Motomasa Suzuki, Kazuo Okanari, Mariko Kasai, Osamu Mitani, Tomoyuki Nakazawa, Taku Omata, Konomi Shimoda, Yuichi Abe, Yoshihiro Maegaki, Kei Murayama, Yuka Murofushi, Hiroaki Nagase, Akihisa Okumura, Yasunari Sakai, Hiroko Tada, Masashi Mizuguchi, Japanese Pediatric Neuro-COVID-19 Study Group, Tsuyoshi Matsuoka

**Affiliations:** Division of Child Neurology and Child Psychiatry, Okinawa Prefectural Nanbu Medical Center and Children’s Medical Center, Okinawa, Japan; Division of Pediatric Critical Care Medicine, Matsudo City General Hospital, Chiba, Japan; Department of Pediatrics, Nagasaki University Hospital, Nagasaki, Japan; Division of Neurology, Saitama Children’s Medical Center, Saitama, Japan; Department of Pediatric Neurology, Fukuoka Children’s Hospital, Fukuoka, Japan; Department of Neurology, Tokyo Metropolitan Children’s Medical Center, Tokyo, Japan; Department of Pediatrics and Adolescent Medicine, Tokyo Medical University, Tokyo, Japan; Department of Pediatrics, Aichi Medical University School of Medicine, Nagakute, Aichi, Japan; Ibaraki Pediatric Education and Training Station, University of Tsukuba, Ibaraki, Japan; Department of Pediatrics, Kurashiki Central Hospital, Okayama, Japan; Department of Pediatrics, Yokohama Rosai Hospital, Kanagawa, Japan; Department of Pediatric Neurology, Shizuoka Children’s Hospital, Shizuoka, Japan; Division of Neuropediatrics, Nagano Children’s Hospital, Nagano, Japan; Department of Pediatrics, Sapporo City General Hospital, Hokkaido, Japan; Department of Pediatrics, Faculty of Medicine, University of Yamanashi, Yamanashi, Japan; ^1^Department of Brain and Neurosciences, Tokyo Metropolitan Institute of Medical Science, Tokyo, Japan; ^2^Department of Pediatrics and Pediatric Neurology, Tokyo Women’s Medical University Yachiyo Medical Center, Tokyo, Japan; ^3^Department of Pediatrics, Jichi Medical University, Tochigi, Japan; ^4^Department of Pediatrics, Japanese Red Cross Kyoto Daiichi Hospital, Kyoto, Japan; ^5^Department of Neurology, Gunma Children’s Medical Center, Gunma, Japan; ^6^Department of Pediatric Neurology, Aichi Children’s Health and Medical Center, Aichi, Japan; ^7^Department of Pediatrics, Oita Prefectural Hospital, Oita, Japan; ^8^Department of Pediatrics, Saitama Citizens Medical Center, Saitama, Japan; ^9^Department of Pediatrics, Fukuyama City Hospital, Hiroshima, Japan; ^10^Department of Pediatrics, Tokyo Metropolitan Toshima Hospital, Tokyo, Japan; ^11^Division of Child Neurology, Chiba Children’s Hospital, Chiba, Japan; ^12^Department of Pediatrics, The University of Tokyo Hospital, Tokyo, Japan; ^13^Division of Neurology, National Center for Child Health and Development, Tokyo, Japan; ^14^Division of Child Neurology, Institute of Neurological Sciences, Faculty of Medicine, Tottori University, Tottori, Japan; ^15^Center for Medical Genetics, Department of Metabolism, Chiba Children’s Hospital, Chiba, Japan; ^16^Department of Pediatrics, Kobe University Graduate School of Medicine, Hyōgo, Japan; ^17^Department of Pediatrics, Aichi Medical University School of Medicine, Nagakute, Aichi, Japan; ^18^Department of Pediatrics, Graduate School of Medical Sciences, Kyushu University, Fukuoka, Japan; ^19^Division of Pediatrics, Chibaken Saiseikai Narashino Hospital, Chiba, Japan; ^20^Department of Pediatrics, National Rehabilitation Center for Children with Disabilities, Tokyo, Japan

**Keywords:** infection-triggered encephalopathy syndrome (ITES), acute encephalopathy with biphasic seizures and late reduced diffusion (AESD), COVID-19, SARS-CoV-2, outcome, clinico-radiological syndrome, acute encephalopathy syndrome

## Abstract

**Background and objectives:**

To clarify whether severe acute respiratory syndrome coronavirus 2 (SARS-CoV-2) infection cause acute encephalopathy in children and which are the most common syndromes that cause them and what are the outcomes.

**Methods:**

A nationwide web-based survey among all members of the Japanese Society of Child Neurology to identify pediatric patients aged < 18 years who developed acute encephalopathy in Japan between 1 January 2020 and 31 May 2022 associated with severe acute respiratory syndrome coronavirus 2 (SARS-CoV-2) infection confirmed by polymerase chain reaction or antigen tests using pharyngeal swabs. Acute encephalopathy was defined as acute onset of impaired consciousness lasting > 24 h or an altered mental state; neurological symptoms arising within 2 weeks of onset of COVID-19 or multisystem inflammatory syndrome in children (MIS-C)/pediatric inflammatory multisystem syndrome (PIMS); evidence of SARS-CoV-2 infection; and reasonable exclusion of other diseases. Patients were divided into the known clinico-radiological acute encephalopathy syndrome group and unexplained or unclassifiable acute encephalopathy group. Outcomes were assessed by pediatric cerebral performance category (PCPC) score at hospital discharge.

**Results:**

Of the 3,802 society members, 217 representing institutions responded, and 39 patients with suspected acute encephalopathy were reported, of which 31 met inclusion criteria. Of these patients, 14 were diagnosed with known clinico-radiological acute encephalopathy syndromes, with acute encephalopathy with biphasic seizures and late reduced diffusion (five patients) being the most common. Five developed acute encephalopathy associated with MIS-C/PIMS. Among 31 patients, 9 (29.0%) had severe sequelae or died (PCPC ≥ 4). Two of three patients with encephalopathy with acute fulminant cerebral edema and two with hemorrhagic shock and encephalopathy syndrome died. The PCPC scores were higher in the known clinico-radiological acute encephalopathy syndrome group than in the unexplained or unclassifiable acute encephalopathy group (*P* < 0.01).

**Discussion:**

Acute encephalopathy related to SARS-CoV-2 infection was demonstrated to be more severe than that caused by other viruses in Japan. Acute encephalopathy syndromes characterized by specific neuroradiological findings was associated with poor clinical outcomes.

## 1. Introduction

At present, the coronavirus disease 2019 (COVID-19) pandemic caused by severe acute respiratory syndrome coronavirus 2 (SARS-CoV-2) remains prevalent. Although the primary site of infection of SARS-CoV-2 is the respiratory system, with no evidence of direct central nervous system invasion, various neurological complications of COVID-19 have been reported (Ellul et al., 2020; Iadecola et al., 2020). Acute encephalopathy is known as a neurological complication of COVID-19, in addition to cerebrovascular disease, encephalitis, meningitis, anosmia/ageusia, and Guillain–Barré syndrome (Ellul et al., 2020; Iadecola et al., 2020). A study has reported that one of the most prevalent neurological syndromes caused by SARS-CoV-2 is acute encephalopathy, which was identified in 1,845 of 3,740 adult patients (49%) hospitalized with COVID-19 (Chou et al., 2021).

Acute encephalopathy triggered by infectious diseases has been widely reported, mainly in children, and most of these cases occur with common viral infections such as influenza virus and human herpes virus-6 (Mizuguchi et al., 2007). Patients present with various degrees of consciousness disorders soon after infection, which may occasionally be accompanied by seizures. Many cases have been reported from Japan and other East Asian countries, and Japanese children may be more susceptible to acute encephalopathy associated with viral infections (Hoshino et al., 2012; Kasai et al., 2020).

Therefore, we conducted a nationwide survey in Japan to determine the current status of pediatric acute encephalopathy associated with COVID-19. Herein, we report on the high incidence of severe, occasionally fatal, acute encephalopathy syndromes associated with COVID-19 in Japanese children.

## 2. Materials and methods

Children aged < 18 years who developed COVID-19-associated acute encephalopathy between 1 January 2020 and 31 May 2022 were included in the study. Acute encephalopathy was defined as a condition meeting all of the following four diagnostic criteria:

(1) acute onset of impaired consciousness (Glasgow Coma Scale < 11 or Japan Coma Scale > 20, lasting > 24 h) or altered mental state such as abnormal speech, behavior, or personality change (Mizuguchi et al., 2021);
(2) neurological symptoms arising in the acute phase (within 2 weeks from onset) of COVID-19 or COVID-19-associated multisystem inflammatory syndrome in children (MIS-C)/pediatric inflammatory multisystem syndrome (PIMS) (Jiang et al., 2020; Whittaker et al., 2020);
(3) SARS-CoV-2 infection confirmed by PCR or antigen tests using pharyngeal swabs; and
(4) reasonable exclusion of other diseases.

We conducted a web-based survey from June 7 to 30, 2022 among all members of the Japanese Society of Child Neurology. The collected data included age of onset, sex, provisional clinical diagnosis, vaccination history against SARS-CoV-2, and outcomes, which were provided by pediatric neurologists at the survey.

We defined five clinico-radiological acute encephalopathy syndromes associated with viral infection: acute encephalopathy with biphasic seizures and late reduced diffusion (AESD); encephalopathy with acute fulminant cerebral edema; acute necrotizing encephalopathy (ANE); hemorrhagic shock and encephalopathy syndrome (HSES); and mild encephalitis/ encephalopathy with a reversible splenial lesion (MERS) as shown in Supplementary Table 1 (Levin et al., 1983; Krishnan et al., 2021; Mizuguchi et al., 2021). We excluded encephalopathy secondary to apparent hypoxia or cerebrovascular disease, metabolic or toxic encephalopathy, acquired demyelinating syndromes, and autoimmune encephalitis. We also excluded patients with underlying genetic abnormalities that may affect the development of encephalopathy. Clinical variables were compared between the known clinico-radiological acute encephalopathy syndrome group and the unexplained or unclassifiable acute encephalopathy group. The severity of respiratory impairment was classified as mild, moderate-1, moderate-2, or severe according to the Japanese Ministry of Health, Labor and Welfare criteria (Supplementary Table 2; Kato, 2021). MRIs were mostly performed a few days after the onset because there is a delay between the onset of disease and the appearance of characteristic MRI findings in AESD and MERS Outcomes were assessed by pediatric cerebral performance category (PCPC) score (Zaritsky et al., 1995) at hospital discharge. If a patient had underlying disability and recovered to baseline status after the illness, the PCPC score was rated 1.

Some patient details have been submitted for publication as case reports by their treating physicians (Nishimura et al., 2022; Saito et al., 2022).

The Mann–Whitney test was used to determine statistical significance for discrete variables, and the chi square test or the Fisher exact test was used for binary variables. Significance was set at *P* < 0.05. Statistical analyses were performed using the R software, version 4.2.1 (R Project for Statistical Computing).

The study was approved by the Institutional Review Board of Tokyo Metropolitan Institute of Medical Science (#20–28). Informed consent for the presentation of neuroimages was obtained from all the patients or their guardians.

## 3. Results

**FIGURE 1:**
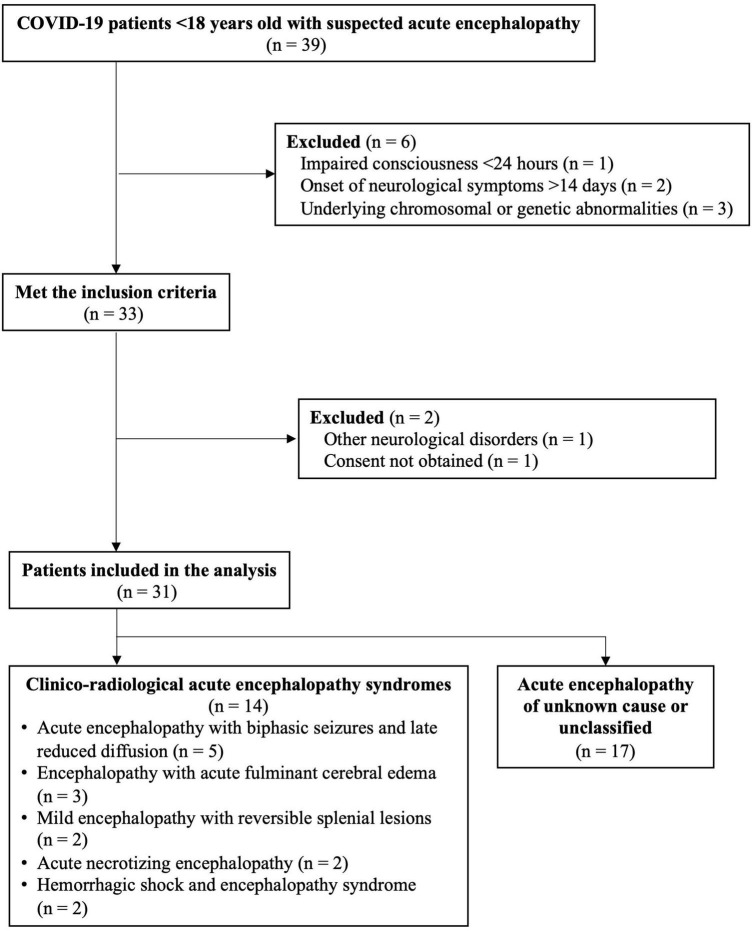
Eligibility flowchart.

Altogether, 3,802 members of the Japanese Society of Child Neurology were surveyed, and 217 responded (response rate: 5.7%) (Figure 1). After excluding duplicates, response was obtained from 201 medical institutions. Of the respondents, 26 (12.0%) reported 39 cases of suspected acute encephalopathy. Six of these cases were excluded because they did not meet the criteria for acute encephalopathy: one because the duration of consciousness impairment was < 24 h, two because the time between infection and the appearance of neurological symptoms exceeded 14 days, and three because underlying chromosomal or genetic abnormalities [1p36 deletion syndrome, *SCN8A* encephalopathy, and *MYRF* variant with recurrent MERS (Saito et al., 2022)]. Hence, 33 patients fulfilled the definition of acute encephalopathy. One patient diagnosed with another immune neurological disease and another who did not provide consent for study participation were excluded. Finally, 31 patients were included in the analysis.

**FIGURE 2:**
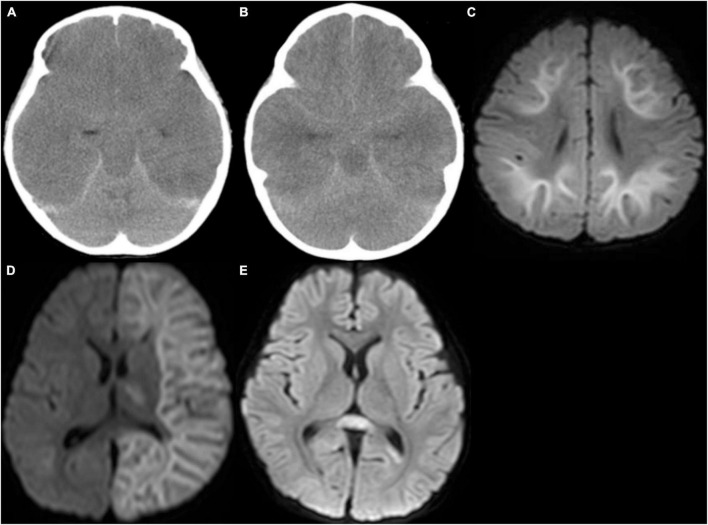
Computed tomography (CT) and magnetic resonance imaging. **(A)** CT in a patient with encephalopathy with fulminant acute cerebral edema revealed diffuse low density in the cerebrum with blurring of gray-white matter junction, and disappearance of ambient cistern, suggesting severe cerebral edema with probable downward herniation. **(B)** CT in a patient with hemorrhagic shock and encephalopathy syndrome revealed severe cerebral edema. **(C)** Diffusion-weighted image in a patient with acute encephalopathy with biphasic seizures and late reduced diffusion (AESD) revealed high signal lesion in the subcortical white matter with sparing peri-Rolandic regions, so-called bright tree appearance. **(D)** Diffusion-weighted image of a patient with unilateral AESD revealed left-sided bright tree appearance (BTA) with a small lesion in the left ventral thalamus. **(E)** Diffusion-weighted image of a patient with mild encephalitis/encephalopathy with reversible splenial lesion (MERS) revealed a high signal lesion in the splenium of the corpus callosum.

Of these, 14 were diagnosed with a known clinico-radiological acute encephalopathy syndrome (Figure 2). The causative diseases were AESD (Takanashi et al., 2006) (five patients), encephalopathy with fulminant acute cerebral edema (Krishnan et al., 2021) (three patients), MERS (Tada et al., 2004) (two patients), ANE (Mizuguchi et al., 1995) (two patients), and HSES (Levin et al., 1983) (two patients). The remaining 17 patients had acute encephalopathy of unexplained or unclassifiable cause.

A summary of the patient data is presented in Table 1. The median age of onset was 5 years (28 days–14 years), with a male-to-female ratio of 13:18. In Japan, Alpha and Delta strains were prevalent until December 2021, and the majority of patients had Omicron strains from January 2022 onward; 29 of the 31 patients developed the disease after January 2022. None of the patients were hospitalized prior to the onset of acute encephalopathy. Neurologic symptoms appeared 0–8 days (median 0 days) after the onset of COVID-19 that was defined as the appearance of either fever or respiratory symptoms, with main initial symptoms being seizures (*n* = 15), impaired consciousness (*n* = 8), and abnormal speech or behavior (*n* = 7). All patients presented these symptoms during the acute encephalopathic phase. Respiratory symptoms at the onset of neurological symptoms were mild in all patients, and no case was preceded by dyspnea due to pneumonia or other respiratory diseases. Only one patient had been vaccinated. Of the 31 patients, five developed acute encephalopathy associated with MIS-C/PIMS, and two of which developed MERS. Nineteen patients recovered to their pre-symptomatic state (PCPC = 1), one with mild disability (PCPC = 2), two with moderate disability (PCPC = 3), three with severe disability (PCPC = 4, including one with pre-existing developmental delay), two in a coma or vegetative state (PCPC = 5), and four died (PCPC = 6). In total, 10 (29.4%) had severe sequelae or died (PCPC ≥ 4) among the 31 patients.

**TABLE 1:** Characteristics and outcomes of pediatric patients with COVID-19-related acute encephalopathy.

	All (*n* = 31)	Group A: Clinico-radiological acute encephalopathy syndromes (*n* = 14)	Group B: Acute encephalopathy of unknown cause or unclassified (*n* = 17)	Group A vs. group B
Age range (median)	0–13 years (5)	0–10 years (4)	0–13 years (5)	NS*
Sex, male (%)	13 (43.8%)	6 (42.9%)	7 (41.1%)	*P* = 1.00
Vaccinated (%)	1 (3.2%)	0 (0%)	1 (5.9%)	*P* = 1.00
Onset after 2022 (%)	29 (93.5%)	13 (92.9%)	16 (94.1%)	*P* = 1.00
**Presenting symptom (%)**				
Seizure	15 (48.4%)	8 (61.5%)	7 (41.1%)	*P* = 0.48
Decreased consciousness level	8 (25.8%)	5 (35.7%)	3 (17.6%)	*P* = 0.41
Abnormal behavior	7 (22.6%)	1 (7.1%)	6 (35.3%)	*P* = 0.094
MIS-C/PIMS (%)	5 (16.1%)	2 (14.2%)	3 (17.6%)	*P* = 1.00
Outcome (%)				*P* < 0.01*
PCPC = 1	19 (61.3%)	5 (35.7%)	14 (82.3%)	
PCPC = 2	1 (3.2%)	0 (0 %)	1 (5.9%)	
PCPC = 3	2 (6.5%)	1 (7.1%)	1 (5.9%)	
PCPC = 4	3 (9.7%)	3 (21.4%)	0 (0%)	
PCPC = 5	2 (6.5%)	1 (7.1%)	1 (5.9%)	
PCPC = 6	4 (12.9%)	4 (28.6%)	0 (0%)	

Examined using the chi-square test or Fisher exact test except for *age and outcome with the Mann–Whitney *U* test. NS, not significant; MISC/PIMS, multisystem inflammatory syndrome in children/pediatric inflammatory multisystem syndrome; PCPC, pediatric cerebral performance category.

When patients were divided into two groups: the known clinico-radiological acute encephalopathy syndrome group (group A: *n* = 14) and the unexplained or unclassifiable acute encephalopathy group (group B: *n* = 17), the PCPC score was higher in group A than in group B (3.50 ± 2.06 vs. 1.41 ± 1.03, *P* < 0.01 by Mann–Whitney *U* test).

When group A was further classified into five clinico-radiological syndromes (Table 2), three patients developed encephalopathy with acute fulminant cerebral edema with two fatal outcomes and two patients developed HSES with fatal outcomes, suggesting that the prognosis for these two syndromes is particularly poor. Two patients with AESD and one with ANE had a PCPC score of 4, while all patients with MERS had a PCPC score of 1.

**TABLE 2:** Outcomes in different acute encephalopathy syndromes.

Clinical syndromes	PCPC score					
	**1**	**2**	**3**	**4**	**5**	**6**
Clinico-radiological acute encephalopathy syndromes (*n* = 14)	5	0	1	3	1	4
Acute encephalopathy with biphasic seizures and late reduced diffusion (*n* = 5)	2	0	1	2	0	0
Encephalopathy with acute fulminant cerebral edema (*n* = 3)	0	0	0	0	1	2
Acute necrotizing encephalopathy (*n* = 2)	1	0	0	1	0	0
Hemorrhagic shock and encephalopathy syndrome (*n* = 2)	0	0	0	0	0	2
Mild encephalopathy with reversible splenial lesions (*n* = 2)	2	0	0	0	0	0
Acute encephalopathy of unknown cause or unclassified (*n* = 17)	14	1	1	0	1	0

PCPC, pediatric cerebral performance category.

## 4. Discussion

In this nationwide surveillance in Japan, 217 out of 3,802 members of the Japanese Society of Child Neurology reported 31 patients with acute encephalopathy. The response rate was low because we asked the representative of the facility to answer, and the membership included many pediatric practitioners and non-physician healthcare professionals. Twenty-nine of the 31 patients developed acute encephalopathy in 2022. By the end of 2021, approximately 240,000 patients aged < 20 years had COVID-19, while in 2022, approximately 1.98 million patients were recorded in a 5-month period. The number of patients with acute encephalopathy in each period was approximately proportional to the overall number of patients with COVID-19. The rapid increase in pediatric acute encephalopathy in 2022 may reflect this infection situation.

Acute encephalopathy associated with COVID-19 reportedly occurs in the elderly in association with respiratory insufficiency. Among 707 patients infected by the SARS-CoV-2, 31 (4.4%) developed acute encephalopathy (64.6 ± 12.1 years). COVID-19 encephalopathy started 20.9 ± 8.1 days after COVID-19 symptom onset, and 28 of 31 (90%) patients had an acute respiratory distress syndrome (Uginet et al., 2021). In the present cohort of Japanese children, respiratory symptoms were mild in all patients, making respiratory compromise as the cause of the encephalopathy unlikely. Therefore, COVID-19-related acute encephalopathy observed in children is considered a different condition from that observed in older hospitalized patients. Moreover, neurological symptoms were the primary manifestation in these cases. To the best of our knowledge, no large case series on pediatric COVID-19 with predominant neurological manifestations has been conducted.

Meanwhile, several case series on acute encephalopathy complicated by COVID-19 in children have been reported. In a UK cohort, neurological manifestations were identified in 52 cases among 1,334 children and adolescents hospitalized with COVID-19, and 25 of them had encephalopathy (Ray et al., 2021). Another study has reported that 365 (22%) of 1,695 patients hospitalized with COVID-19 had documented neurologic involvement, among which 43 (12%) developed life-threatening conditions, including 15 with severe encephalopathy (LaRovere et al., 2021). In addition, at least 18 patients with acquired demyelinating syndromes were reported as neurological complications of COVID-19, of which NMOSD was the most common (*n* = 7) (Feizi et al., 2022).

Some infection-triggered acute encephalopathies are characterized by a stereotypical clinical course and specific neuroimaging findings, and thus we recognized these as specific clinico-radiological syndromes and analyzed the cases of acute encephalopathy from this perspective (Mizuguchi et al., 2007). Such infection-triggered encephalopathy syndromes include encephalopathy with fulminant acute cerebral edema (Krishnan et al., 2021), HSES (Levin et al., 1983), AESD (Takanashi et al., 2006), ANE (Mizuguchi et al., 1995), or MERS (Tada et al., 2004). In the present cohort, these clinico-radiological syndromes correlated with a poorer prognosis compared to those in the unclassified acute encephalopathy group.

Some of these acute encephalopathy syndromes have a hyperacute onset and are often difficult to rescue due to rapidly progressive brain edema and consequent loss of brain function. All four deaths in this study were characterized by fatal brain edema. Of note, encephalopathy with fulminant acute cerebral edema, often fatal due to rapidly progressive brain swelling, has been reported (Krishnan et al., 2021; LaRovere et al., 2021). This encephalopathy, observed in previously healthy children, may be relatively common in COVID-19. Two of four fatal cases met the diagnostic criteria for HSES. Most of the HSES cases reported in the past were infants, and similarities with heat stroke have been suspected (Bacon and Hall, 1992). However, since all of our two cases were older children and inappropriate temperature control was not reported, the case reported here as HSES may be a different disorder from conventional HSES. The two encephalopathy syndromes with acute cerebral edema are the most critical ones associated with COVID-19. Although the cause of this rapidly progressive brain edema is unknown, we postulate a disruption of the blood-brain barrier due to excessive uncontrolled inflammation. The involvement of cytokines and chemokines in the hemorrhagic shock and encephalopathy syndrome associated with viral infections other than COVID-19 has been demonstrated, (Yamaguchi et al., 2021) and interestingly, this is consistent with recent observations in COVID-19-related encephalopathy (Pilotto et al., 2020).

In addition to the four deaths, five patients, including two with AESD and one with ANE, were left with severe or profound disability (PCPC = 4 or 5). ANE is a severe encephalopathy characterized by symmetrical involvement of thalami, putamina, cerebral white matter, or brainstem (Mizuguchi et al., 1995). Familial ANE caused by *RANBP2* gene mutations is known (Neilson et al., 2009), although the majority of cases in Japan are sporadic. AESD is a syndrome of clustered brief seizures and characteristic magnetic resonance imaging (MRI) diffusion-weighted imaging findings several days after initial prolonged status epilepticus (Takanashi et al., 2006). AESD is characterized by frontal lobe predominant lesions, which may suggest that the virus has an affinity for specific areas of the brain. In support of this, there is some evidence that SARS-CoV-2 could preferentially and directly target the frontal lobes (Toniolo et al., 2021). It is the most frequent acute encephalopathy syndrome in Japan and has been reported almost exclusively in Japan (Kasai et al., 2020). Some genetic susceptibility has been postulated, and a polymorphism of *STK39* gene has recently been identified (Kasai et al., 2022).

COVID-19-related acute encephalopathy in children is often associated with MIS-C/PIMS. Splenial signal change is often observed in COVID-19-related MIS-C/PIMS (Abdel-Mannan et al., 2020; Lindan et al., 2021). MIS-C/PIMS is associated with 48% of pediatric COVID-19-related neurologic complications, of which 22 (46%) had acute encephalopathy and seven had signal changes in the splenium of the corpus callosum (SCC) (Ray et al., 2021). SCC lesions associated with viral infections, termed MERS, generally have a good prognosis, although not necessarily when associated with COVID-19. The few cases associated with MIS-C/PIMS and the majority of cases with encephalopathy in the acute phase of COVID-19 infection were characteristic of our cohort.

As described above, COVID-19 was associated with various types of acute encephalopathy syndromes. This is similar, for example, to the acute encephalopathy syndrome associated with influenza, indicating that different viruses cause the same clinical phenotype (Mizuguchi et al., 2007). These observations can be explained by the hypothesis that a non-specific immune response to viral infection may trigger acute encephalopathy syndromes. In general, these infection-triggered encephalopathy syndromes have no evidence of virus infiltration in the brain and are considered to be different from primary encephalitis caused by a neurotropic virus.

Previous reports of virus-related acute encephalopathy outcomes from Japan indicated that PCPC = 1 in 56%, PCPC = 2 or 3 in 14%, PCPC = 4 or 5 in 12%, and PCPC = 6 in 5% (Kasai et al., 2020). Compared with these results, COVID-19-related acute encephalopathy may have a relatively poor prognosis, that is, 9/31 patients (29.0%) had severe sequelae or died (PCPC ≥ 4). The type of acute encephalopathy syndrome varied partly according to the causative virus: in influenza-associated encephalopathy, MERS (25%) and AESD (16%) predominated, while encephalopathy with fulminant acute cerebral edema (0.5%) and HSES (0%) were rare. The relatively high proportion of encephalopathy with fulminant acute cerebral edema and HSES in COVID-19-related acute encephalopathy syndromes may be associated with poor outcomes.

This study had several limitations. First, due to its retrospective nature, no standardized protocols for diagnosis and treatment were considered. Although clinical practice guidelines for acute encephalopathy syndromes are followed in Japan, different treatment strategies may have affected outcomes. Second, infection control measures often precluded MRI, which may have resulted in underdiagnosis of acute encephalopathy syndromes. Thirdly, detailed clinical course and laboratory data were not collected in the present survey and require further study in the future. Finally, the different methods used in the present and previous studies made it difficult to precisely compare the data between them.

## 5. Conclusion

COVID-19-related acute encephalopathy syndromes are characterized by a rapid onset of seizures and impaired/altered consciousness without severe respiratory symptoms and by potentially poor outcomes. These represent an important public health threat in children, and SARS-CoV-2 should be recognized as a pathogen causing acute encephalopathy syndrome.

## Data availability statement

The original contributions presented in this study are included in the article/Supplementary material, further inquiries can be directed to the corresponding author.

## Ethics statement

The studies involving human participants were reviewed and approved by the Institutional Review Board of Tokyo Metropolitan Institute of Medical Science (#20–28). Written informed consent to participate in this study was provided by the participants or their legal guardian/next of kin.

## Japanese Pediatric Neuro-COVID-19 Study Group

Division of Child Neurology and Child Psychiatry, Okinawa Prefectural Nanbu Medical Center and Children’s Medical Center, Okinawa, Japan: Tsuyoshi Matsuoka. Division of Pediatric Critical Care Medicine, Matsudo City General Hospital, Chiba, Japan: Hiroshi Oakada. Department of Pediatrics, Nagasaki University Hospital, Nagasaki, Japan: Tatsuharu Sato. Division of Neurology, Saitama Children’s Medical Center, Saitama, Japan: Kenjiro Kikuchi. Department of Pediatric Neurology, Fukuoka Children’s Hospital, Fukuoka, Japan: Satoshi Akamine. Department of Neurology, Tokyo Metropolitan Children’s Medical Center, Tokyo, Japan: Nanako Kawata. Department of Pediatrics and Adolescent Medicine, Tokyo Medical University, Tokyo, Japan: Shinichiro Morichi. Department of Pediatrics, Aichi Medical University School of Medicine, Nagakute, Aichi, Japan: Hideyuki Iwayama. Ibaraki Pediatric Education and Training Station, University of Tsukuba, Ibaraki, Japan: Ryuta Tanaka. Department of Pediatrics, Kurashiki Central Hospital, Okayama, Japan: Yoshiyuki Hanaoka. Department of Pediatrics, Yokohama Rosai Hospital, Kanagawa, Japan: Yuki Minamisawa. Department of Pediatric Neurology, Shizuoka Children’s Hospital, Shizuoka, Japan: Tatsuya Ema. Division of Neuropediatrics, Nagano Children’s Hospital, Nagano, Japan: Mitsuo Motobayashi. Department of Pediatrics, Sapporo City General Hospital, Hokkaido, Japan: Tomoshiro Ito. Department of Pediatrics, Faculty of Medicine, University of Yamanashi, Yamanashi, Japan: Fumikazu Sano.

## Author contributions

HS, JT, and MM: concept and design. HS, JT, OM, KaM, MS, KO, MK, HK, KS, and TS: acquisition, analysis, and or interpretation of data. HS and JT: drafting of the manuscript. HS: statistical analysis, obtained funding, had full access to all the data in the study, takes responsibility for the integrity of the data, and the accuracy of the data analysis. AO and MM: supervision. All authors contributed to the critical revision of the manuscript for important intellectual content.
