# Supplementary material for: Severe pediatric acute encephalopathy syndromes related to SARS-CoV-2
Source: Front Neurosci. 2023 Feb 27;17:1085082. doi: 10.3389/fnins.2023.1085082 (PMC10008884; doi:10.3389/fnins.2023.1085082)
Supplement: Supplementary file 1 [file Data_Sheet_1.docx]

**Supplementary Table**

**Supplementary Table 1** Definition for infection-triggered acute encephalopathy syndromes

| **Definition for acute encephalopathy** |
| --- |
| A clinical presentation of altered mental status, defined as decreased (Glasgow Coma Scale <11) or altered level of consciousness, lethargy, or personality change, lasting for >24 hours |

| **Diagnostic criteria of acute encephalopathy with biphasic seizures and late reduced diffusion (AESD)**  A diagnosis of AESD is made if there is any of 3 to 5 in addition to 1 and 2.  Clinical findings:   1. AESD occurs in children during the course of a febrile infection. Other conditions, such as head trauma, child abuse, hypoxic encephalopathy, other encephalopathy syndromes and encephalitis, are excluded. 2. The onset is on the day of fever or the next day, with a febrile seizure (early seizure) usually lasting longer than 30 min. 3. On day 3 to 7, secondary seizures (late seizures) most often in a cluster of complex partial seizures) or deterioration of consciousness level.   Image findings:   1. Diffusion-weighted image shows high signal in the subcortical white matter (bright tree appearance) and/or in the cortex at day 3 to 14. Sparing of pre- and postcentral gyrus (central sparing) is usually observed. 2. After 2 weeks, residual lesions or atrophy on CT/MRI, or decreased blood flow on SPECT are observed in the frontal or fronto-parietal region, often with central sparing.   Reference findings:   1. HHV-6 and influenza virus are the most frequent causative agents. 2. After an early seizure, consciousness level improves. 3. CT and MRI performed on days 1 and 2 are normal. 4. Prognosis varies from mild intellectual disability to severe psychomotor impairment. |
| --- |

| **Diagnostic criteria of encephalopathy with acute fulminant cerebral edema**   1. Definition for encephalitis 2. Age ≥6 months 3. Immunocompetent status 4. Encephalopathy (depressed or altered level of consciousness lasting 24 hours, lethargy, or change in personality)   along with at least 1 additional finding:   1. Fever 2. Seizure 3. Focal neurologic deficit 4. Cerebrospinal fluid pleocytosis 5. Electroencephalographic changes consistent with encephalopathy 6. Neuroimaging suggestive of encephalitis 7. Progression to diffuse cerebral edema on neuroimaging and/or autopsy 8. No other recognized etiology for cerebral edema (eg, organic, metabolic, toxin) |
| --- |

| **Diagnostic criteria of acute necrotizing encephalopathy (ANE)**   1. Acute encephalopathy related to a febrile viral infection: rapid reduction of consciousness and seizures. 2. Cerebrospinal fluid examination shows normal cell counts and increased protein concentration. 3. Symmetrical and multiple brain lesions on head CT and/or MRI. Bilateral thalamic lesions are always observed. Lesions are often found also in periventricular white matter, internal capsule, putamen, upper brainstem tegmentum, and cerebellum. No lesions in the other areas. 4. Elevated serum transaminase levels with no elevation in serum ammonia levels. 5. Exclusion of other diseases: Severe bacterial and viral infections, fulminant hepatitis, toxic shock, hemolytic uremic syndrome, Reye syndrome, hemorrhagic shock and encephalopathy syndrome, and heat stroke, mitochondrial disorders such as Leigh encephalopathy, glutaric acidemia, methylmalonic acidemia, infantile bilateral striatal necrosis, Wernicke encephalopathy, carbon monoxide poisoning, acute diffuse encephalomyelitis, acute hemorrhagic leukoencephalopathy, angiitis, arterial and venous infarction, hypoxia and traumatic head injury. |
| --- |

| **Diagnostic criteria of hemorrhagic shock and encephalopathy syndrome (HSES)**  A child under 16 years of age with acute onset of:   1. Encephalopathy 2. Shock 3. DIC 4. Diarrhea (may be bloody) 5. Falling hemoglobin concentration and platelet counts 6. Acidosis 7. Raised hepatocellular enzymes 8. Renal function impairment 9. Negative cultures of blood and cerebrospinal fluid 10. Definite HSES: all nine criteria satisfied. 11. Probable HSES: either: (i) eight criteria satisfied but one not met or (ii) at least seven criteria satisfied but no information on the remainder. 12. Indeterminate or not HSES: either: (i) insufficient information, or (ii) another diagnosis more likely, or (iii) presence of a preceding event which may have distorted the clinical. |
| --- |

| **Diagnostic criteria of mild encephalitis/encephalopathy with a reversible splenial lesion (MERS)**  Clinical findings:   1. Delirious behavior, consciousness disturbance or seizures within 1 week after fever. 2. Recovery without sequelae within 1 month after onset of neurological symptoms. 3. Exclusion of other neurological diseases such as ADEM, AESD and acute cerebellar inflammation. 4. Neurological symptoms persisting for more than 12 h.    Note: Delirious behavior may be intermittent.  Imaging findings:   1. DWI showing a reversible splenial lesion with homogenously reduced diffusion with mild T1 and T2 signal abnormalities. 2. Lesion involving at least the splenium. It may expand to the entire corpus callosum or symmetrical white matter. 3. Disappearance of lesion within 2 months, leaving no abnormal signal or atrophy. |
| --- |

**Supplementary Table 2** Severity classification of COVID-19 by Japanese Ministry of Health, Labour and Welfare

| Severity | Oxygen saturation | Clinical state |
| --- | --- | --- |
| Mild | SpO_2_ ≥ 96% | No respiratory symptoms  Coughing only; no shortness of breath |
| Moderate I | 93% < SpO_2_ < 96% | Shortness of breath and pneumonia findings |
| Moderate II | SpO_2_ ≤ 93% | Oxygen administration required |
| Severe |  | Admission to ICU or mechanical ventilator required |

ICU = intensive care unit; SpO_2_ = oxygen saturation of peripheral artery

**Supplementary Table 3** Japan Coma Scale for grading impaired consciousness

| Grade | Consciousness Level |
| --- | --- |
| 1-digit code | The patient is awake without any stimuli, and is: |
| 1 | Almost fully conscious |
| 2 | Unable to recognize time, place, and person |
| 3 | Unable to recall name or date of birth |
| 2-digit code | The patient can be aroused (then reverts to previous state after cessation of stimulation): |
| 10 | By easily by being spoken to (or is responsive with purposeful movements, phrases, or words) |
| 20 | With a loud voice or shaking of shoulders (or is almost always responsive to very simple words like yes or no or to movements) Only by repeated mechanical stimuli |
| 30 | With a loud voice or shaking of shoulders (or is almost always responsive to very simple words like yes or no or to movements) Only by repeated mechanical stimuli |
| 3-digit code | The patient cannot be aroused with any forceful mechanical stimuli, and: |
| 100 | Responds with movements to avoid the stimulus |
| 200 | Responds with slight movements, including decerebrate and decorticate posture |
| 300 | Does not respond at all except for changes in respiratory rhythm |
